# A New Spectrum of Self-Injuries: TikTok-Linked Lesions

**DOI:** 10.7759/cureus.58226

**Published:** 2024-04-14

**Authors:** Leila Laghmiche, Gwendy Dupire, Diane Franck

**Affiliations:** 1 Surgery, Université Libre de Bruxelles, Brussels, BEL; 2 Dermatology, Hôpitaux Universitaires de Bruxelles, Brussels, BEL; 3 Plastic and Reconstructive Surgery, Hôpitaux Universitaires de Bruxelles, Brussels, BEL

**Keywords:** general dermatology, pediatric hand surgery, pediatric dermatology, burn injury, plastic and reconstructive surgery

## Abstract

The end of childhood and adolescence are two critical periods. Patients’ immaturity in making decisions can lead to irreversible health consequences. The use of social media exposing children to a wide variety of content may result in dangerous behavior. This has been seen with the emergence of many challenges such as the “Eraser Challenge,” “Salt Ice Challenge,” and “Benadryl Challenge.” Here, we describe two cases of dermatologic lesions linked to social media challenges.

## Introduction

TikTok® is a short-form video mobile application developed by the Chinese company Byte Dance® and released in 2016. The user can submit videos lasting 15 seconds to 10 minutes. TikTok has been criticized for having inappropriate content that may result in not only misinformation and psychological effects but also addiction and serious injuries. Some dangerous viral trends such as the “Benadryl Challenge” and “Black-out Challenge” started on TikTok and led to the hospitalization and death of children [1-3]. It is crucial to draw the attention of medical staff to these new types of pathologies associated with the utilization of social networks. Given their burgeoning evolution, there is a noticeable surge in deviations linked to their usage, including health-related implications. Here, we report two cases of a nine-year-old and a 15-year-old girl who tried challenges related to videos seen on TikTok.

## Case presentation

Case 1

A nine-year-old girl was admitted to the emergency department after she tried to spray an entire bottle of deodorant approximately 1 cm away from the skin surface of her left hand.

**Figure 1:**
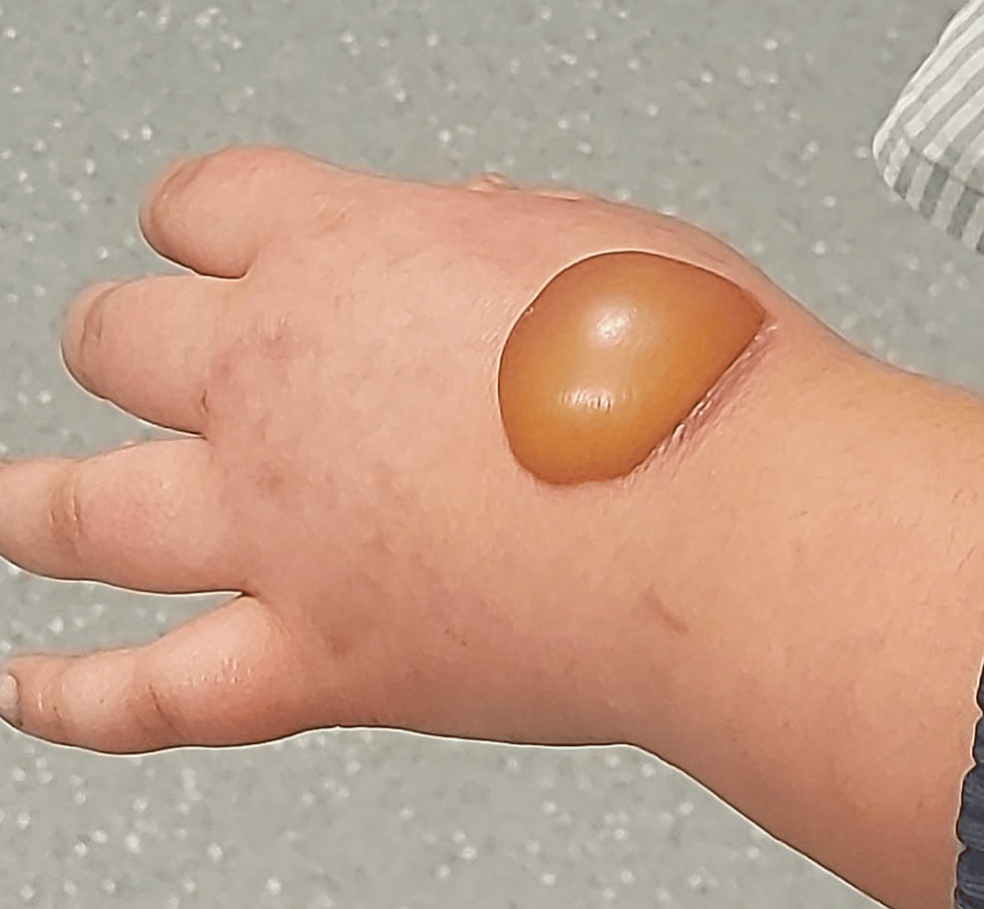
A second-degree circular burn with a phlycten.

**Figure 2:**
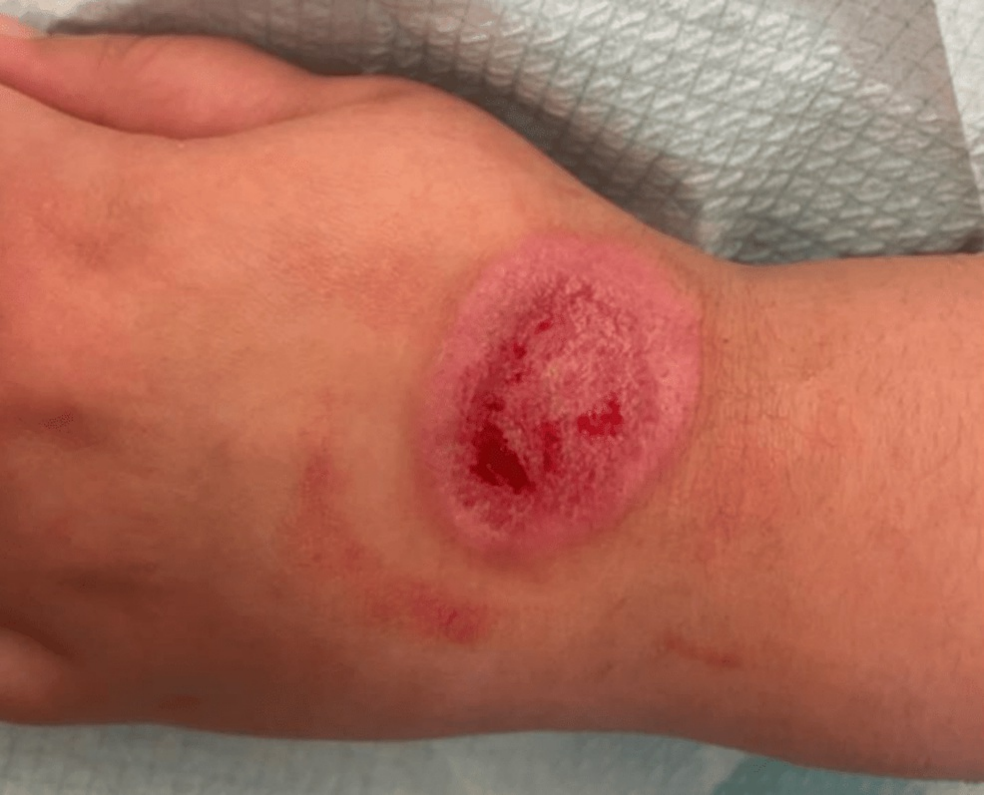
The well-healing wound.

**Figure 3:**
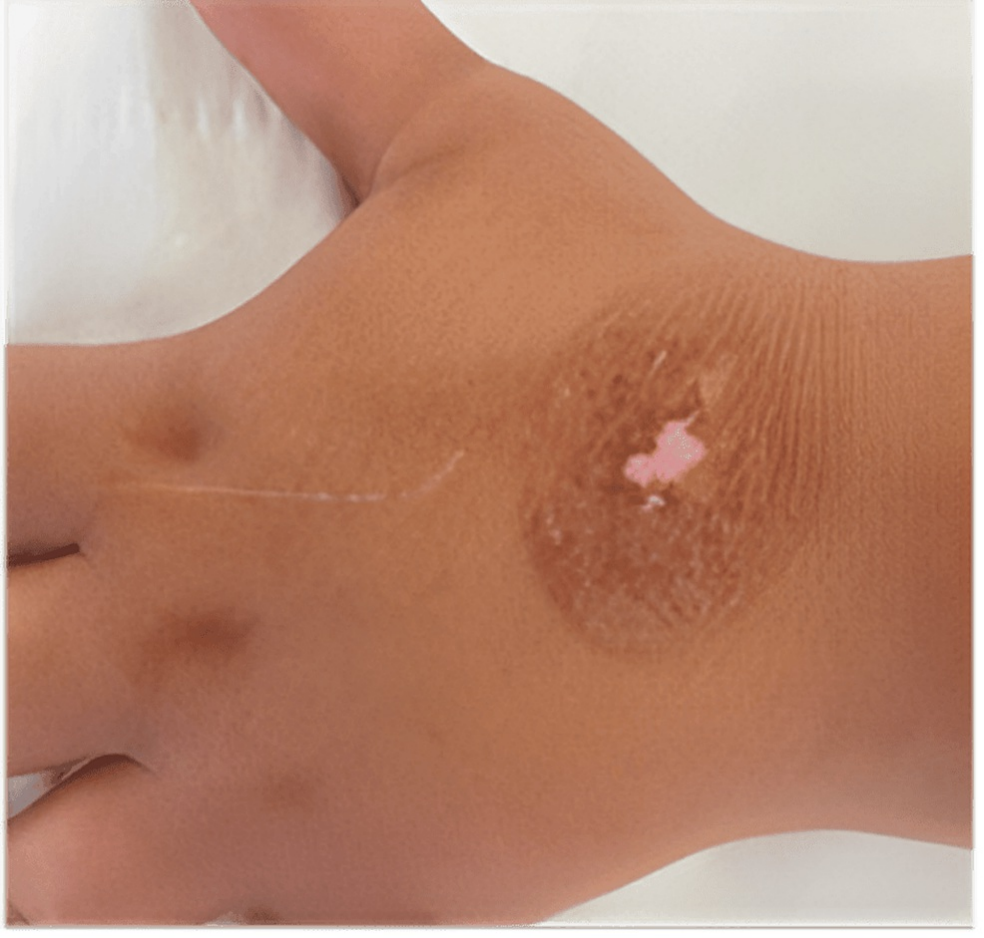
Hyperpigmented scar.

The physical examination showed a second-degree circular burn measuring 4 × 3 cm on her left hand due to the cooling of the bottle’s content, as illustrated in Figure 1. She saw this pain resistance challenge on TikTok. The burn was treated as a thermal burn with Flammacerium® (cerium nitrate-silver sulphadiazine) once every two days. She was followed up for 42 weeks every two days, and then once a week for another two weeks. The burn evolved well, as shown in Figure 2, leading to a dyschromic and non-hypertrophic scar after three months, as shown in Figure 3.

Case 2

**Figure 4:**
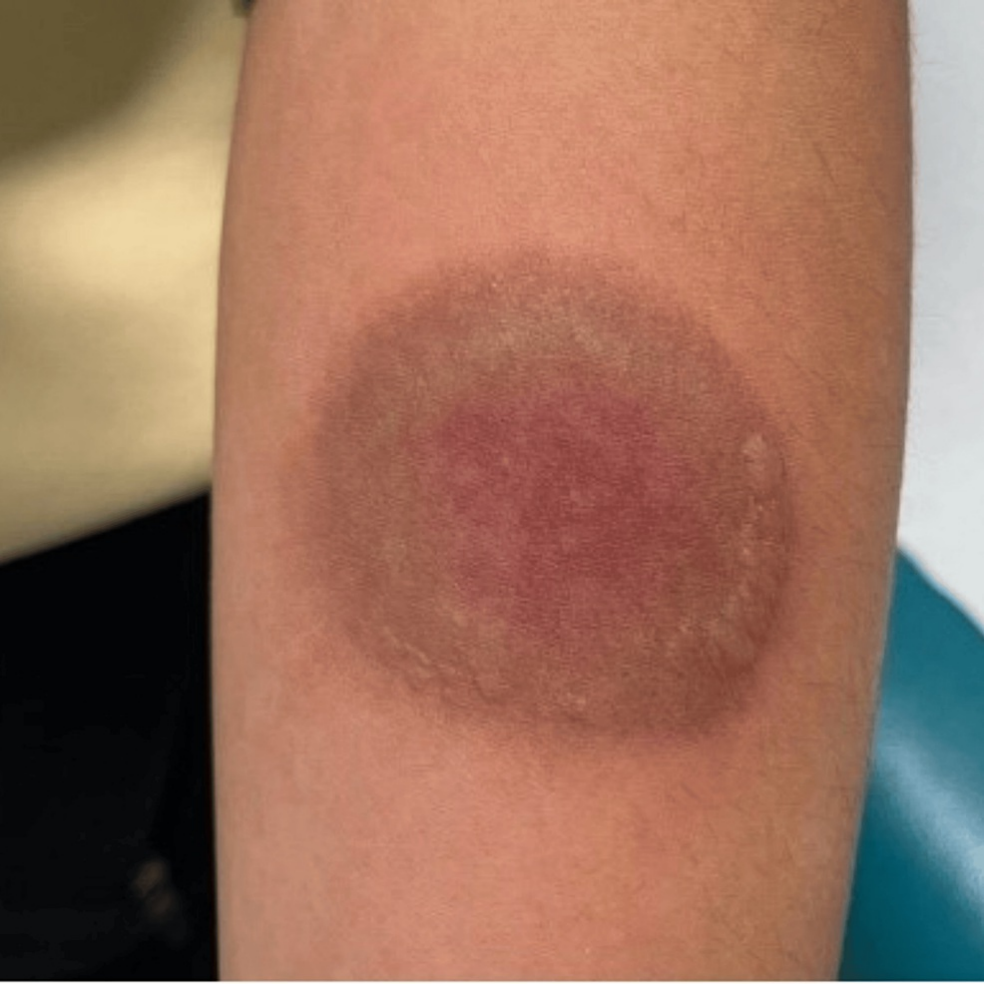
Aspect of the lesion in the emergency department.

A 15-year-old girl presented to the emergency department with a spontaneous circular lesion measuring 2.5 × 3.2 cm on the left forearm. The lesion had an erythematous-violaceous center and a well-defined grayish border, as shown in Figure 4.

**Figure 5:**
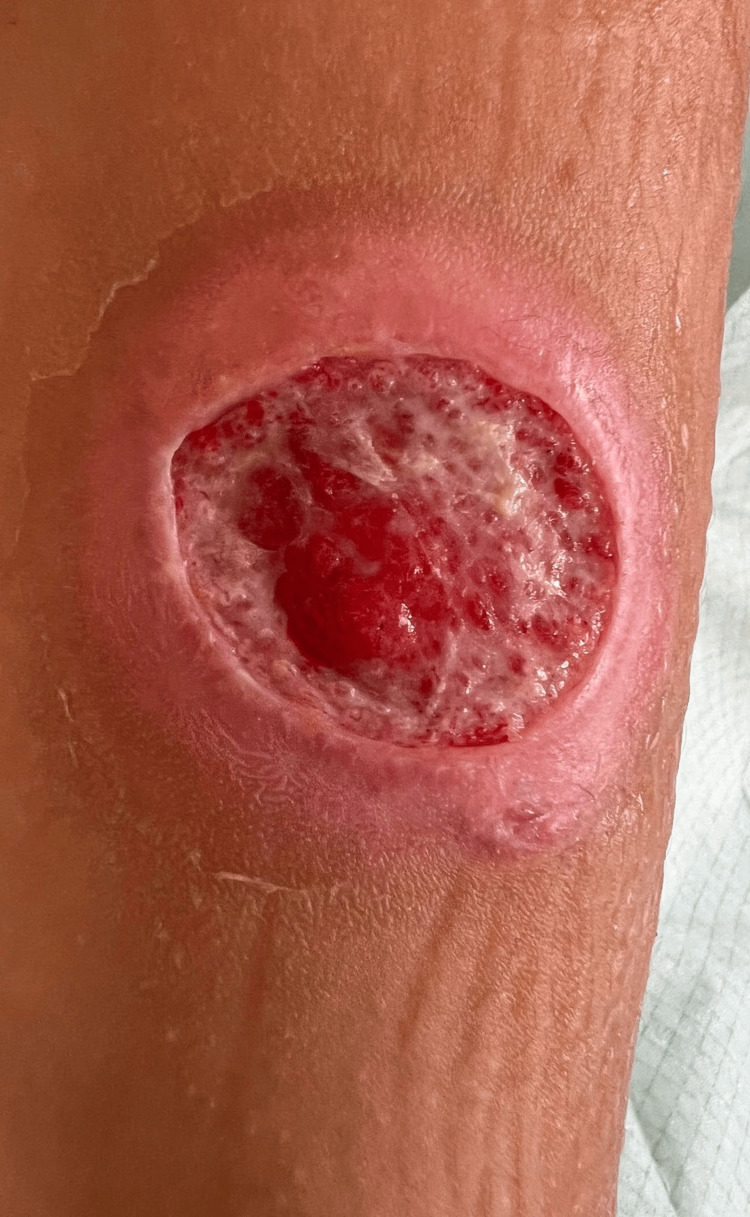
The lesion after two weeks of treatment.

There was no history of insect bites, recent respiratory infections, use of medications, recent travel, or allergies. The blood test showed no inflammatory syndrome and was within the normal range. After a few days, the periphery detached superficially and was followed by a much deeper detachment of the central part, leaving a well-defined ulceration, as shown in Figure 5. A dermatological pathomimicry was suspected and a biopsy was performed. A differential diagnosis of pyoderma gangrenosum was excluded by the biopsy.

**Figure 6:**
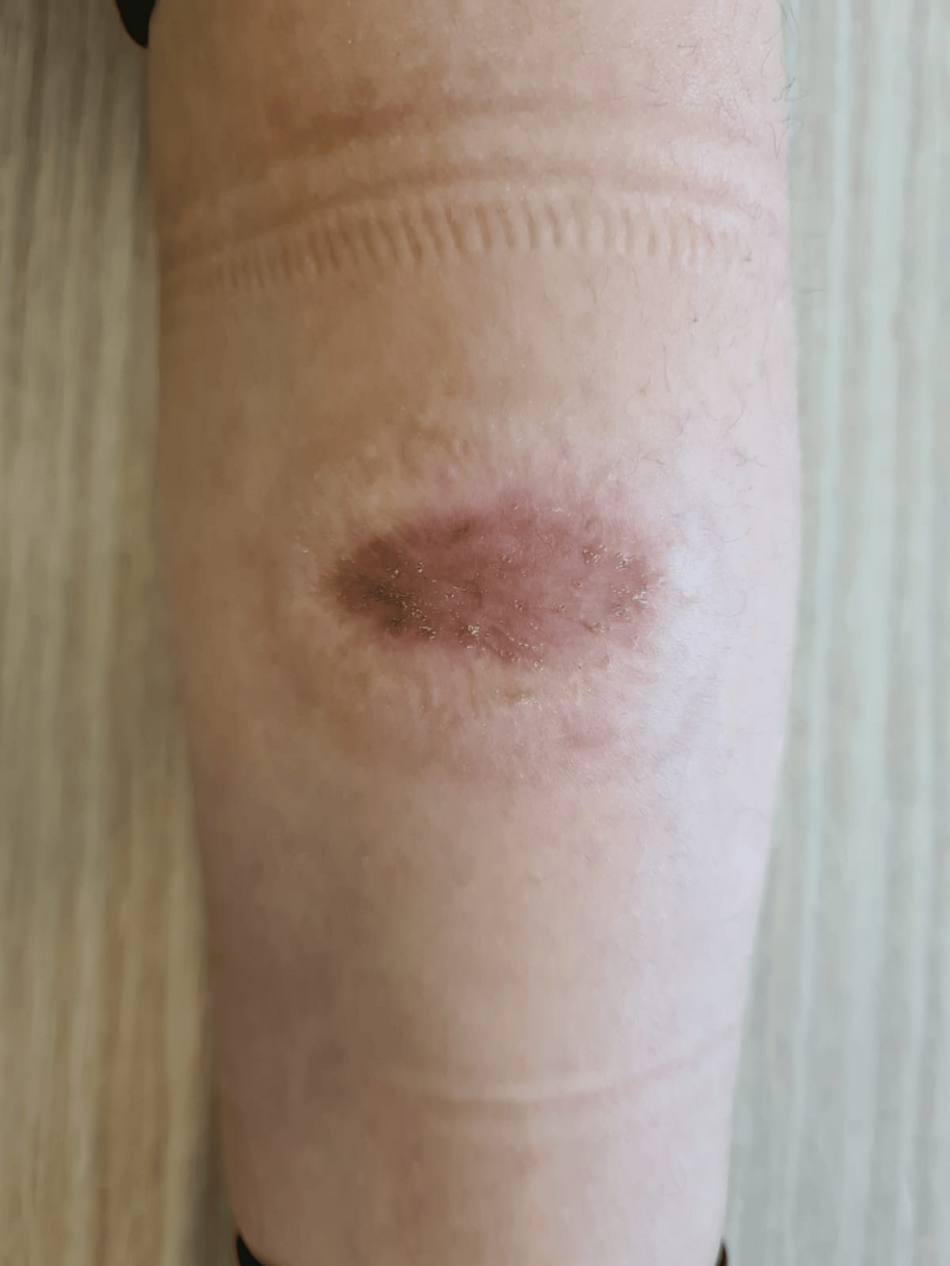
Six months after skin grafting.

The lesion was first treated conservatively with algino gel and then with a fusidic acid cream. After three weeks of conservative treatment, as the lesion was not healing well, a thin skin graft was performed. The patient finally admitted that the injury was caused by a dry cupping challenge seen on TikTok. The skin graft healed well and the patient was seen after six months with a satisfying result, as illustrated in Figure 6.

## Discussion

Social media has undoubtedly emerged as a powerful tool for disseminating information and educating the younger demographic on crucial health topics [4]. Studies have even demonstrated its efficacy in reducing preoperative anxiety, showcasing its potential for positive impact in healthcare settings [5]. However, recent reports have shed light on a concerning trend: the emergence of new types of injuries and burns stemming from the misuse of social media platforms and their content, particularly among vulnerable populations [1-3].

It is imperative to raise awareness about this novel spectrum of injuries associated with social media challenges. It is also crucial to acknowledge the potential challenges in obtaining a complete medical history due to patient discomfort or embarrassment. These lesions, often linked to viral challenges circulating on social media platforms, exhibit distinct characteristics that can aid clinicians in their identification [6].

For example, the “Deodorant Challenge,” which results in cold burns. Clinically, these burns typically manifest as circular lesions located on the non-dominant arm, accompanied by phlyctens, and tend to resolve spontaneously over time. On the other hand, while dry cupping has gained popularity in recent years, there is currently a lack of literature documenting skin necrosis associated with this practice.

Clinicians should be vigilant for certain telltale signs when encountering these injuries. For instance, a solitary erythematous-violaceous circular lesion on the non-dominant arm that fails to improve despite appropriate treatment should raise suspicion for a lesion linked to dry cupping.

The unfamiliarity and underestimation of these new phenomena can lead to delays in effective management and, consequently, poor patient outcomes. Moreover, the oversight of these injuries may result in unnecessary investigations, further increasing healthcare costs. Therefore, healthcare providers must stay informed about emerging trends on social media and be equipped to recognize and address associated health risks promptly and effectively.

Efforts to educate both healthcare professionals and the general public about the potential dangers of these social media challenges are paramount in preventing such injuries and promoting overall well-being.

## Conclusions

The lack of familiarity and the tendency to underestimate the emerging phenomenon of social media-related health risks can contribute to delays in effective management, ultimately leading to suboptimal outcomes. Moreover, this oversight may inadvertently drive up healthcare costs due to the need for additional and often unnecessary investigations. Thus, enhancing awareness and understanding of these novel health risks associated with social media is critical to mitigating their impact on patient care and healthcare expenditures.
